# Comparing Current and Future Land Suitability for Growing Rainfed Corn (*Zea mays*) in Georgia, USA

**DOI:** 10.3390/plants13172486

**Published:** 2024-09-05

**Authors:** Ruth Kerry, Ben Ingram, Connor S. Golden

**Affiliations:** 1 Geography Department, Brigham Young University, Provo, UT 84602, USA; 2 Facultad de Ingeniería, Universidad de Talca, Camino a Los Niches Km. 1, Curicó 3344158, Chile

**Keywords:** land suitability, climate change, corn, spatial distribution

## Abstract

Land suitability (LS) classifications are essential for efficient and sustainable agricultural land use. With climate change, future LS classifications are necessary to ensure that crop growth remains sustainable and prevents land degradation. This study develops a current LS classification for rainfed corn (*Zea mays*) growth in the state of Georgia, USA, which is validated using historical census data on yield, acres planted, and corn crop lost. Significant (*p* < 0.05) differences were found between yield, acres planted, and crop loss percentage across LS classes for many years. Soil factors (Ph and soil texture) showed significant differences in fewer years compared to climate and topography factors, as soil factors can be altered by management practices such as liming and irrigation. Future LS classes determined by climate factors indicated a shift to the northwest of 150–300 km by the year 2100 based on the RCP4.5 or RCP8.5 emissions scenarios. The northwards shift in more suitable land due to rising maximum temperatures is expected to limit rainfed corn growth in Georgia in the future. As urban areas become more suitable for corn growth, farmers may need to plant crops earlier, irrigate, or switch to different crops. These results have important implications for agricultural planning and policy in the state of Georgia.

## 1. Introduction

From the 1950s to the 1970s, “reconnaissance land resources surveys” were conducted in the developing world [1] to promote more efficient and sustainable land management. However, the need for a unified system of land evaluation to facilitate information exchange between countries that had developed different systems was recognized [1]. The FAO framework for land evaluation was developed in 1976 [2]. One of its six basic principles was that land suitability (LS) refers to use on a sustained basis, ensuring that land will not be degraded by a particular use [1]. Prior to this framework, the USDA Land Capability Classification [3] was the most widely used system, categorizing soil into eight general land uses (four for arable crops, and others including pasture, woodland, and recreation) to indicate the most appropriate use of fields/land that could be sustained and not lead to land degradation [1]. Since the inception of the FAO Land Suitability framework [2], LS assessments have been widely carried out in both developed and developing countries, with a need for system revision identified in 2007 [1].

LS classifications show whether the climate, topography, and soil factors in a given area are “suitable” for growing certain crops in irrigated or rainfed settings without land degradation. Classifications identifying agricultural land uses as suitable compared to other land uses have appeared in the literature [4,5,6]. These studies seem to relate more to the USDA Land Capability Classification [3]. However, recently several LS classifications for growing a range of specific crops such as maize [7,8], wheat and barley [9], wheat and maize [10], rice [11], rapeseed [12,13], and tobacco [14] have also appeared in the academic literature. LS classifications for semi-arid areas [10,13] such as parts of China [14,15,16] and Iran [6,8,9,13,17] have received particular attention in the literature, as detailed LS classifications have previously been lacking in these areas. Additionally, there is a need to investigate how to expand crop production sustainably in these marginal areas so that land degradation does not occur. More detailed LS studies have become possible in recent years with the use of geographic information systems to combine data layers [8,10,12,14,17,18,19,20,21,22,23] and the use of remotely sensed data [5,19,22,23] to inform on soil characteristics and crop health based on current land uses. The use of machine learning approaches [6,24] have also facilitated the identification of patterns in large quantities of data.

Much academic research and many standard biogeography textbooks have documented how the natural distributions of plant species have changed with the major climate changes in the Quaternary period [25,26]. As an important part of LS for growing given crops is based on climate, expected future changes are likely to alter the zones where certain crops may be successfully and sustainably grown [27,28]. Globally, future LS for growing wheat [29,30] and soybeans [31] has been investigated. Future suitability for growing soybeans [15] and cotton [16] has been investigated within China and the Xinjiang Province of China, respectively. However, likely within-state shifts in the LS for growing key crops in the USA has not received detailed study. This is important to investigate, as along with shifts in the zones of LS for particular crops, the abundance of insect [32] and fungal [33] pests changes as climate alters. In addition, it is likely that highly carcinogenic aflatoxins produced by fungi will increasingly contaminate crops in a warmer future, leading to more crop disposal as FDA legislative limits [34] are exceeded more often [35,36,37].

The research in this paper investigates likely shifts in LS classes for rainfed corn (*Zea mays*) under future climate change scenarios in the state of Georgia, USA. This work will help determine if where corn is grown should shift or whether other adaptation strategies should be employed to sustain yield in the future. While it is desirable to have an overall or multi-criteria based LS classification that integrates soil, topographic, and climatic factors, such as those produced by Grassano et al. [12], Zolekar [19], Mendra & Delali [38], Ostovari et al. [13], and Mustafa [23], the FAO [1] notes that “Factorial approaches to land suitability that provide a single numerical index derived from addition, multiplication or normalization of component factors” can be “misleading”. The FAO [1] also notes how the “pseudo-accuracy of one numerical value often masks methodological problems such as how to weight and combine individual factors into a single scale, or the subjective expert judgment of individual weightings and dependencies”. Consequently, as the likely influence of future climate on LS classes is investigated here, the contributions of climatic, soil, and topographic factors are purposefully kept separate in this paper. This will allow spatial analysis of shifts in the most suitable areas for corn growth based on different future climate characteristics.

This paper develops a GIS-based LS classification for current rainfed maize growth in the state of Georgia, USA. LS classes are determined for climate, topographic, and soils data based on the criteria of Tashayo et al. [8]. The current LS classification is validated by comparing historical county-level agricultural census data on acres planted, yield, and the percentage crop loss for the LS zones. Historical insurance indemnity payments for crop losses from various sources are also used to assess the validity of the current LS classification. To evaluate the likely influence of future climate patterns on the LS of the state of Georgia, USA for rain-fed corn growth, LS classes based on future climate projections and the same soil and topographic information are developed for 30-year intervals until 2100. Two emissions scenarios are considered, the RCP4.5 [39] and RCP 8.5 [40] (Representative Concentration Pathway). The RCP4.5 scenario assumes that emissions policies will cause stabilization in emissions by 2100 [39]. The RCP8.5 scenario assumes “business as usual” with no policies to stabilize emissions [40]. The spatial distribution of LS classes at each time step is analyzed, and shifts in LS zones in GA under different potential future climates are investigated.

## 2. Methods

### 2.1. Climate Data

**Figure 1:**
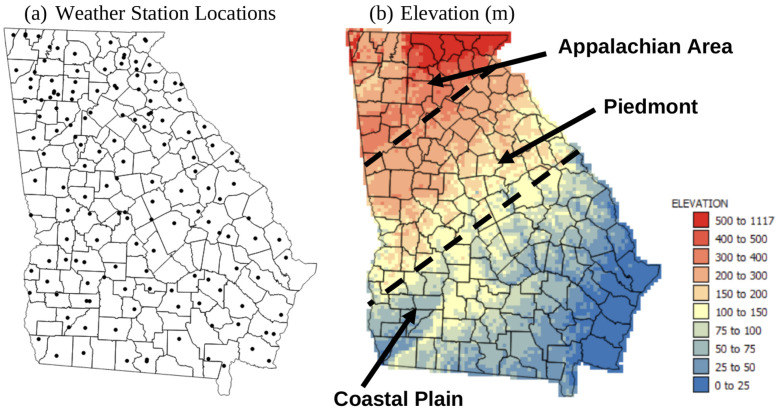
Maps showing: (**a**) weather station locations (black dots) and county boundaries (black lines) within Georgia, and (**b**) elevation in meters above sea level for the state of Geogia. Thin black lines are county boundaries, and thick dashed lines show simplified boundaries of main landform settings. Adapted from https://georgiastudies.gpb.org/c2-s2, accessed on 10 June 2024.

Annual and monthly climate normals (1991–2020) of mean, minimum, and maximum temperatures (Tmean, Tmin, and Tmax) and precipitation for each weather station in Georgia (see Figure 1a for locations) were extracted from https://www.ncei.noaa.gov/access/us-climate-normals/ (accessed on 1 May 2021), and were kriged to county centroids and a 5 km grid. There were 88 stations that had temperature data and 102 that had precipitation data available for the state of GA. LS classes were assigned based on the climate criteria listed in Table 1, which were adapted from Tashayo et al. [8].

### 2.2. Topography Data

Topography in the state of Georgia can be split into three main areas (Figure 1b). Elevations in the southern Georgia coastal plain are at or below sea level and range up to 700 m in the north at the edges of the Appalachian plateau. A 90 m digital elevation model was extracted from the SRTM (Shuttle RADAR Topography Mission) dataset at https://earthexplorer.usgs.gov/ (accessed on 1 May 2021). Average elevation and slope for each 5 km grid cell was calculated using SAGA GIS version 7.8.2 [41]. The dominant elevation and slope values for each county were assigned to county centroids. LS classes based on topographic criteria were assigned to each 5 km grid square and county centroid based on the criteria in Table 1.

### 2.3. Soils Data

Shapefiles of soil types were downloaded for each county from the Soil Survey Geographic Database (SSURGO) of the Natural Resources Conservation Service (NRCS), USDA at https://www.nrcs.usda.gov/resources/data-and-reports/soil-survey-geographic-database-ssurgo (accessed on 1 May 2021). Shapefiles were sampled at each location on a 5 km grid and the pH and soil texture were extracted for each grid cell and assigned LS classes based on the information in Table 1. Dominant soil attribute values and associated LS classes were also assigned to country centroids based on the criteria in Table 1.

### 2.4. Corn Yield Data

Agricultural census data for each county on corn yield, acres planted, and the percentage differences in acres planted and harvested (% crop loss) were extracted from https://www.nass.usda.gov/Statistics_by_State/Georgia/Publications/County_Estimates/index.php (accessed on 1 May 2021) for 2018–2020 and from https://www.nass.usda.gov/Quick_Stats/index.php for 1954–2018 (accessed on 1 May 2021). These were analyzed in conjunction with historical and future climate normal data to investigate which weather conditions were associated with higher yields, larger numbers of acres planted, and larger percentages of crop loss. In addition, the mean centers of locations with higher than average yield, acres planted, and percentage crop loss (% crop loss) were calculated and plotted on maps to show any patterns in movement of these areas between 1954 and 2020. In Georgia, the main crops that occur in rotation with corn are soybeans, cotton, and peanuts, so future LS work can focus on whether the LS classes for rainfed corn are also appropriate for the crops that are grown in rotation with it.

### 2.5. Agricultural Insurance Indemnity Payment Data

Maps of agricultural insurance payments due to different causes of loss were created in the AgRiskViewer website at https://gallery3.jornada.nmsu.edu/rma/rma-data-viewer.html (accessed on 5 July 2024). These were used along with the historical acres planted, yield, and % crop loss data to validate the current LS classification developed for the state of Georgia.

### 2.6. Statistical Analysis

To validate the current LS classes, historical county level data on acres of corn planted, corn yield, and the % crop loss for each year were compared between LS classes using Mann–Whitney U and Kruskal–Wallis H tests. This was performed using data for 1954–2020 (66 years of data). A comparison test was computed to compare the acres planted, yield, and % crop loss for corn between LS classes for each LS parameter. For example, within the state of Georgia. there are only two land suitability classes for Tmean (S2, moderately suitable and S3, marginally suitable), so Mann–Whitney U tests were used to compare acres planted, yield, and %crop loss between Tmean LS classes. For all other LS parameters, three or more LS classes were present in the state of Georgia, so Kruskal–Wallis H tests were used to compare the acres planted, yield, and % crop loss between LS classes. The results of these 66 comparison tests per LS parameter were reported in terms of the proportion of tests for individual years where a significant difference (*p* < 0.1) in yield, acres planted, or % crop loss was found between LS classes.

### 2.7. Future Climate Data

Following validation of the current LS classification, LS classifications based on future climate data were determined. Projections of annual mean, minimum. and maximum temperatures (Tmean, Tmin, and Tmax) and precipitation for 30 year periods under the RCP 4.5 and RCP 8.5 scenarios (Representative Concentration Pathway) were extracted from https://climatetoolbox.org/tool/Climate-Mapper (accessed on 1 May 2021) for each county centroid in GA. The RCP4.5 scenario is more conservative, where measures to reduce emissions have been implemented, and RCP8.5 is a “business as usual” emissions scenario. County level future climate predictions were then assigned to LS classes based on the climate criteria in Table 1. For each climate variable, the mean centers of each LS class were calculated for each time period to show any trend in movement of the most suitable climates for growing corn within the state of Georgia.

## 3. Results and Discussion

### 3.1. Land Suitability Classes for Topographic and Soil Factors

**Figure 2:**
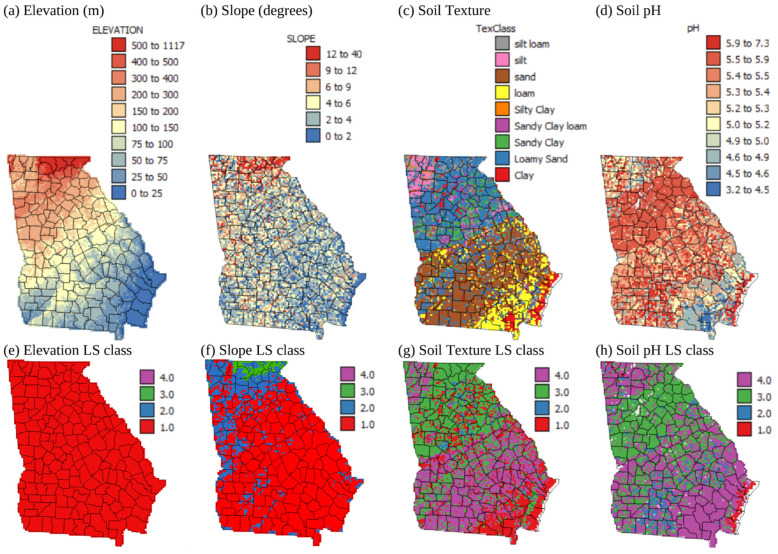
Maps showing (**a**) elevation, (**b**) slope, (**c**) soil texture, and (**d**) soil pH on a 5 km grid and land suitability (LS) class for (**e**) elevation, (**f**) slope, (**g**) soil texture, and (**h**) soil pH. Land suitability classes 1–4 correspond with S1, highly suitable; S2, moderately suitable; S3, marginally suitable; and N4, not suitable (from Table 2).

Figure 2a–d show the values of elevation, slope, soil texture, and pH for the state of Georgia represented on a 5 km grid. Figure 2e–h show the ordinal LS classes that these values translate to, with 1 being highly suitable and 4 being not suitable. The LS classes for each variable are based on the criteria in Table 1, which were adapted from Tashayo et al. [8]. Figure 2a,e show that all areas of Georgia are below 1700 m in elevation and so are highly suitable for corn production. Figure 2b,f show that slopes in the majority of locations in the coastal plain and piedmont regions in the central and southern parts of the state (Figure 1b) are <2%, making them highly suitable for corn growth. There are, however, some areas of 2–6% slope in the southern and central areas of the state. In contrast, the north-west part of the state, or the Appalachian area (Figure 1b), is dominated by steeper slopes, with slopes in this area generally falling into the moderately or marginally suitable LS classes.

Figure 2c shows that the soil textures for the majority of the state of Geogia are very sandy, with sand and loamy sand textural classes dominating in the coastal plain and piedmont areas (Figure 1b), respectively. The coastal plain is the main agricultural area in the state of Geogia, and currently, about 90% of corn crops in Georgia are irrigated to circumvent the problem of sandy soils (https://www.nass.usda.gov/Quick_Stats/; accessed on 13 July 2024). There are, however, large areas of loam and clay soils closer to the coast of Georgia (Figure 2c), where irrigation is not needed. Figure 2g shows that most of the coastal plain area is not suitable for rainfed corn growth based on the sandy soils, and most of the piedmont area is only marginally suitable for rainfed corn growth, again due to the dominance of loamy sand soils (Figure 2c). Most of the areas with soil textures highly suitable for rainfed corn growth are located by the coast (Figure 2g). Finally, soil pH in the state of Geogia ranges from highly acidic to neutral (Figure 2d). pH values < 5.5 make the soils throughout much of the coastal plain and the Appalachian area not suitable for corn growth. The piedmont area is dominated by soils with a pH of 5.5–5.9, making them marginally suitable for corn growth. The only areas in the state of Georgia with pH values that are suitable and highly suitable for corn are small and scattered areas of the coastal plain (Figure 2h). However, this limitation can be overcome, as soil pH can be increased by the application of lime. Liming is actually routinely practiced for corn growth in Georgia [42].

### 3.2. Current Land Suitability Classes Based on Climate

**Figure 3:**
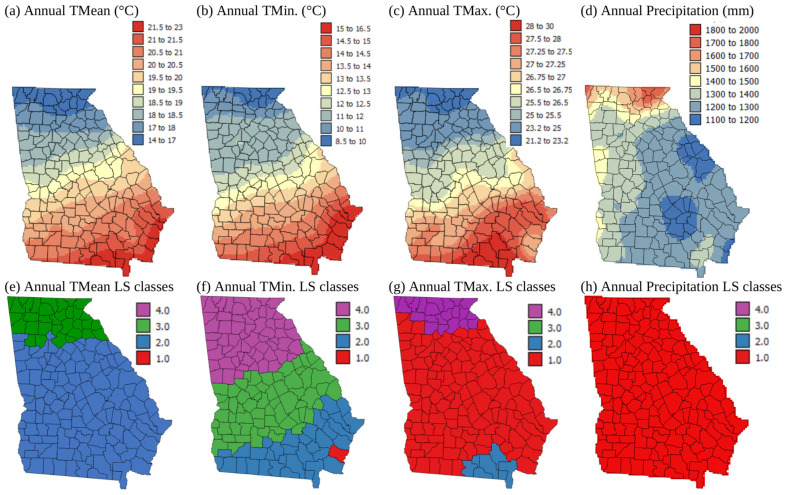
Maps showing kriged 1991–2020 climate normals (**a**–**d**) and current land suitability classes at the county level (**e**–**h**) for rainfed corn (*Zea mays*) in the state of Georgia, USA. Land Suitability Classes 1–4 correspond with S1, highly suitable; S2, moderately suitable; S3, marginally suitable; and N4, not suitable (from Table 2).

Figure 3a–d show climate normals kriged to a 5 km grid throughout the state of Georgia for annual mean, minimum, and maximum temperatures (Tmean, Tmin, and Tmax) and precipitation. Figure 3e–h show how these spatial patterns in climate translate into LS classes at the county level based on the values in Table 1. Tmean and Tmin tend to be slightly lower than ideal for corn growth in the state of Georgia, with the majority of the state being moderately suitable for corn growth in terms of Tmean (Figure 3e). However, in terms of Tmin, the Appalachian and piedmont areas are not suitable and only marginally suitable (Figure 3f). This is probably the reason why corn is currently planted in May in the north of the state, but in March in the south of the state [43]. In terms of Tmax (Figure 3c,g), the Appalachian area is too cool, but the majority of the state has maximum temperatures that are highly suitable for corn growth, except for a small area in the south of the state where Tmax is higher, making that area just moderately suitable for corn growth.

Tashayo et al. [8] do not mention an optimal annual precipitation amount for rainfed corn growth, but Purdue Extension mentions that 600 mm of rainfall is needed for rainfed corn growth [44]. Figure 3d shows that annual rainfall is >600 mm for the whole state. Additionally, the growing season (May–September) precipitation was investigated, and it is greater than 450 mm, the amount needed for high yields, for all counties [44]. The growing season can vary from April to August and May to September depending on planting dates, which are usually in March in Southern Georgia, and harvest dates, which are often in August or September [45]. Based on this, the whole state is categorized as highly suitable in terms of precipitation for rainfed corn growth (Figure 3h). Most of the state is highly suitable for growing corn in terms of topography and precipitation levels, but large parts of the southern areas, where the temperatures are most suitable, have soil texture and pH limitations.

### 3.3. Validation of Current Land Suitability Classes Based on Historical Data

Table 2 shows the proportion of comparison tests for individual years (1954–2020) for which there were significant (*p* < 0.1) differences in terms of yield, acres planted, and the % crop loss (percent difference in acres planted and harvested) between LS classes based on topography, soils, and climate. Table 2 shows that there were significant differences in acres planted, yield, and % crop loss in most years between the current LS zones for each factor. However, the results for slope and soil pH suggest that the slope has little influence on yield and % crop loss and that the pH has little influence on yield. Indeed, the significant differences between LS classes often showed the reverse pattern than expected for soil and slope factors, with higher yields in areas with acidic or light textured, less suitable, soils. These numbers suggest that slope and soil factors are less important to overall LS. Significant differences in acres planted, yield, and % crop loss reflected the expected order with LS classes for climate variables. The expected result was that higher yields, acres planted, and lower % crop loss would be observed for the more suitable LS classes. The effects of soil texture and pH can be managed with irrigation and liming, whereas the effects of climate are harder to manage. Indeed, currently, most corn grown in Georgia is grown in areas with sandy and acidic soils, but approximately 90% of corn crops in Georgia are irrigated to circumvent the problem of sandy soils (https://www.nass.usda.gov/Quick_Stats/ accessed on 13 July 2024).

**Table 2:** Mann–Whitney U and Kruskal–Wallis H test results. Percentage of years (1954–2020) or of 66 tests with significant (*p* < 0.1) difference in acres planted, yield, or % crop loss between current LS classes for each variable and land suitability parameter.

	Land Suitability Parameters					
Variable	Tmean	Tmin	Tmax	Slope	Texture	pH
Acres Planted	99	96	94	58	70	70
Yield	48	82	67	31	48	34
% Crop Loss	73	94	67	25	88	79

Tmean, mean temperature; Tmin, minimum temperature; Tmax, maximum temperature.

A total of 198 Mann–Whitney U tests were computed to compare acres planted, yield, and % crop loss (66 per variable, one per year) between the two Tmean LS classes shown in Figure 3e. For Tmin, Tmax, slope, texture, and pH, Kruskal–Wallis H tests were computed for each year (66) to compare acres planted, yield, and % crop loss between LS classes based on each parameter. Kruskal–Wallis H tests were computed for these LS parameters, as 3–4 LS classes were present in the state of Georgia for each parameter (see Figure 2 and Figure 3). Table 2 shows that Tmin is currently one of the strongest influences on acres planted, yield, and % crop loss, whereas there are less frequent differences between Tmax LS classes in terms of yield and % crop loss. This suggests that in some years, Tmax may be too high and decrease yield and lead to crop loss. A key reason for crop disposal in years with high temperatures could be associated with aflatoxin contamination, which has been shown to be worse in years when June Tmax is high [46,47], and can lead to the disposal of large amounts of crop. Table 2 also suggests that Tmean has less influence on yield than acres planted or % crop loss. Table 2 suggests that Tmin and Tmax are the most important variables for corn LS in Georgia, and that the LS approach is generally useful.

**Figure 4:**
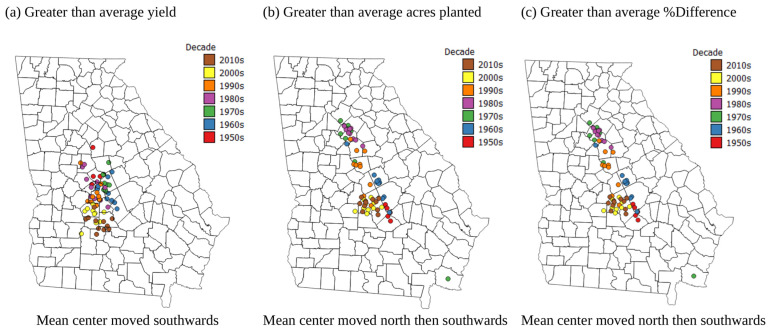
Maps of Georgia showing the mean centers from 1954 to 2020 of (**a**) greater than average yield, (**b**) greater than average acres planted, and (**c**) greater than average % crop loss.

Figure 4a shows that the areas with greater than average corn yield have moved southwards between 1954 and 2020. However, over the almost 70-year study period, the areas with greater than average acres planted and greater than average % crop loss have moved northwards and then back southwards. The initial northwards shift may have been to take advantage of more suitable land in terms of soil texture and pH (see Figure 2g,h), but the southwards shift for acres planted after the northwards shift may be due to expansion of the urban and suburban areas into agricultural lands around Atlanta in the NW of the state. This expansion of urban areas could also be the reason for the southern movement after a northerly movement of the areas with greater than average % crop loss. Indeed, analysis of the historical data showed a major reduction in the acres of corn planted, but a major increase in yield over the 70-year period of the historical data (see Figures S1 and S2 in Supplemental Information). This suggests that as agricultural methods have improved, production may have moved to the locations with the most suitable soils and climate, and could account for the pattern of movement in the mean centers of above average yield, acres planted, and % crop loss (Figure 4).

**Figure 5:**
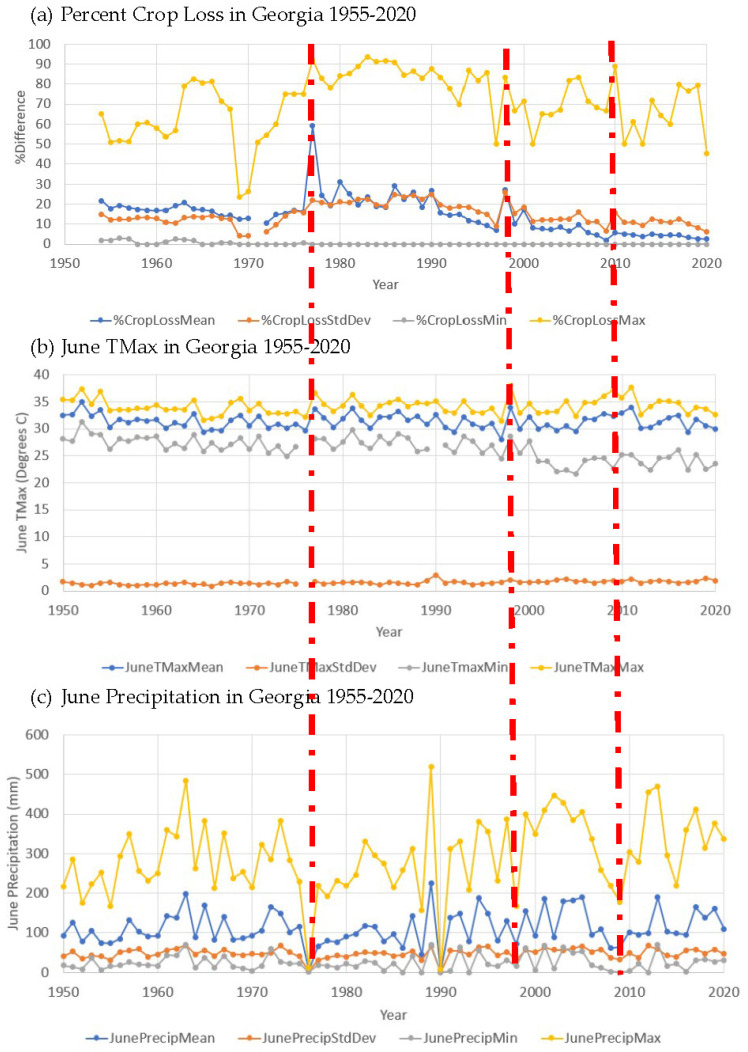
Graphs for 1950–2020 for the state of Georgia showing: (**a**) % crop loss, (**b**) June Tmax, and (**c**) June precipitation. For each variable shown in (**a**–**c**), lines are given for the mean (Mean), standard deviation (StdDev), minimum (Min), and maximum (Max) values for each year. Red dashed lines indicate severe drought years with high percentages of crop loss, high June TMax and low June precipitation.

Figure 5 shows links between peaks in % crop loss, peaks in June Tmax, and troughs in June precipitation. This suggests greater loss of crops during drought years like 1977, 1998, and 2009, shown with red vertical lines in Figure 5. This is particularly the case for droughts that strike during the delicate mid-silk growth stage [46], which tends to occur in June in Georgia, given that corn is mostly planted in March [48]. These drought conditions have also been linked with increased aflatoxin production, which means crops may have to be disposed of for public health reasons [46]. It should also be noted that the droughts identified in Figure 5 are more concentrated later in the study period which could be a sign of a warming trend or increased drought frequency as climate changes.

**Figure 6:**
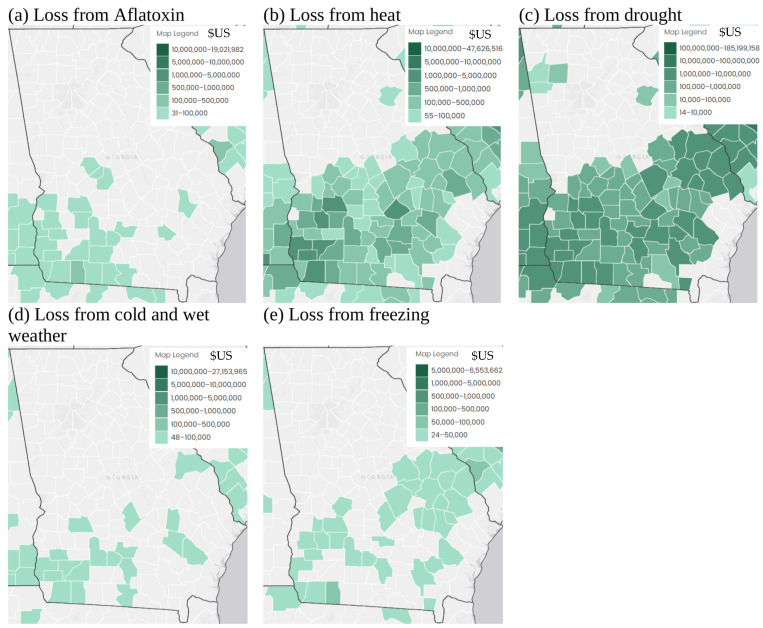
Maps showing insurance indemnity payments in $US for 1998–2022 for loss of corn (*Zea mays*) crops due to (**a**) aflatoxin contamination, (**b**) heat, (**c**) drought, (**d**) cold, wet weather, and (**e**) freezing. Maps created in AgRiskViewer website at https://gallery3.jornada.nmsu.edu/rma/rma-data-viewer.html (accessed on 5 July 2024).

Figure 6 shows insurance indemnity payments for losses of corn crops between 1998 and 2022. The losses from aflatoxin contamination are concentrated in the southwest of the state and tend to be less in dollar amounts (Figure 6a). The losses from cold, wet weather and freezing (Figure 6d,e) are slightly more widespread spatially, but are for similarly low dollar amounts. In contrast, however, the insurance indemnity payments from heat and drought losses are widespread throughout the coastal plain region, which is the main agricultural area in the state of Georgia, and the claims are for far higher dollar amounts, by factors greater than 1000. This suggests that in the last 20 years, warming/drought has been far more of a detrimental issue in agriculture than cold weather or freezing. It also suggests that there may be a warming trend, and there is likely to be a change in climate-based LS classes for the future under different climate change scenarios. Indeed, investigation of the monthly minimum and mean temperatures for May–September for 1950–2020 showed relatively higher minimum and mean temperatures in most months for the 1990–2020 period compared with the 1950–1990 period.

### 3.4. Future Land Suitability Classes Based on Climate Predictions

**Figure 7:**
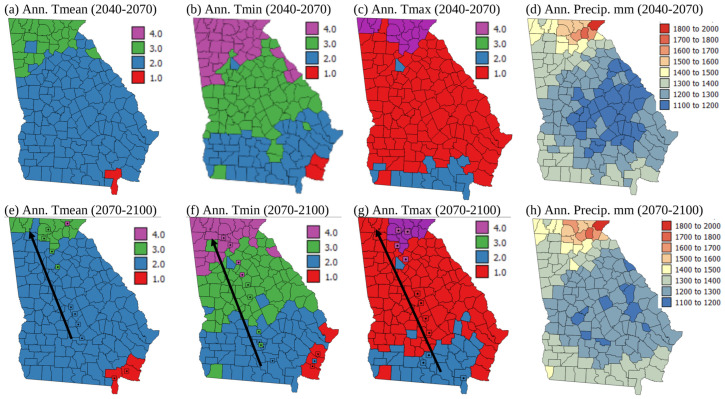
Maps of land suitability (LS) class for rainfed corn (*Zea mays*) growth for climate normals 2040–2070 and 2070–2100 under the RCP 4.5 emissions scenario. Colored squares in (**e**–**g**) show the mean centers of each LS class for 1970–2000, 2010–2040, 2040–2070, and 2070–2100. The black arrow shows the direction of movement of mean centers of LS classes over time. Land suitability classes 1–4 correspond with S1, highly suitable; S2, moderately suitable; S3, marginally suitable; and N4, not suitable from Table 2.

**Figure 8:**
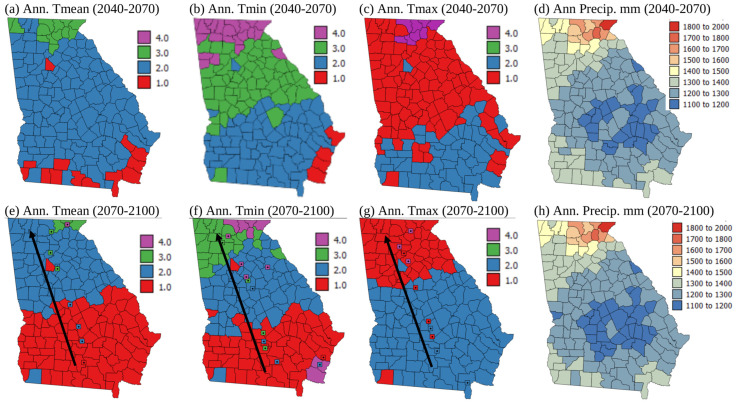
Maps of land suitability (LS) class for rainfed corn (*Zea mays*) growth for climate normals 2040–2070 and 2070–2100 under the RCP 8.5 emissions scenario. Colored squares in (**e**–**g**) show the mean centers of each LS class for 1970–2000, 2010–2040, 2040–2070, and 2070–2100. The black arrow shows the direction of movement of mean centers of LS classes over time. Land suitability classes 1–4 correspond with S1, highly suitable; S2, moderately suitable; S3, marginally suitable; and N4, not suitable (from Table 2).

Figure 7 shows maps of LS zones at the county level for each climate variable for 2040–2070 and 2070–2100 under the RCP4.5 scenario, apart from precipitation, where the precipitation totals are shown rather than LS classes. Historical county level weather data along with future predicted weather are plotted as maps in Supplemental Information Figures S2–S5. In Figure 7 and Figure 8, precipitation patterns by county are shown rather than precipitation LS classes because annual precipitation for all counties is >600 mm, so all counties are highly suitable in terms of precipitation, yet it is useful to see under the different climate change scenarios whether various counties are getting wetter or drier.

Figure 8 shows maps of LS zones at the county level for each climate variable for 2040–2070 and 2070–2100 under the RCP8.5 scenario, apart from precipitation, where the precipitation totals are shown instead. Figures S2–S5 in the Supplemental Information show the temperature maps from which the LS classes in Figure 7 and Figure 8 were derived. They show increases in annual Tmean, Tmin, and Tmax over time throughout the state, and also show that these increases are greatest in 2070–2100 under the RCP8.5 emissions scenario. The bottom row of the maps in Figure 7 and Figure 8 have square icons (the mean centers) and arrows showing how the mean center of each LS class moves due to climate change under each scenario. Figure 7 and Figure 8 show that land becomes more suitable for corn growth in terms of annual Tmin and Tmean over time under both emissions scenarios, with a north-westerly shift in the most suitable zones. This may mean that planting in northern Georgia could occur earlier in the future as currently corn is planted in March in Southern Georgia but mid-May in more northerly parts of the state [48]. Zhu et al. [16] similarly found that suitability zones for cotton, which is frequently grown in rotation with corn in Georgia, would shift northwards in Xinxiang Province, China. Guo et al. [30] showed that due to northward movement in the areas more suitable for wheat growth, the area where wheat will be grown will be reduced in China in a warmer future. Nevertheless, Yue et al. [29] showed that climate change will benefit wheat planting in middle-high latitude locations at the global level. Likely suitability for corn growth globally in a warmer future should be investigated.

In contrast to patterns in Tmin and Tmean, annual Tmax showed a north westerly shift in less suitable zones. These findings are similar to those of Zhu et al. [15], who showed that certain parts of China should see improvements in LS for soybean growth, while other areas will become less suitable. In Figure 7 and Figure 8, the mean centers of each LS class generally show larger shifts to the northwest (200–300 km) under the RCP8.5 scenario than the RCP4.5 scenario (100–150 km). Figure 7 and Figure 8d,h show that the precipitation levels are relatively stable in time and with emissions scenarios. Although Figure 7h shows that the driest counties (1100–1200 mm precipitation) are less abundant in 2070–2100 under the RCP4.5 emissions scenario, in general, there is no major increase or decrease in precipitation expected in Georgia with climate change. The seasonal timing, year to year variability, and intensity of rainfall, however, may shift with climate change. These can all influence the suitability of land for growing corn, but they cannot be reflected in a LS classification that uses annual precipitation totals to determine suitability. Therefore, future LS classifications should incorporate notions of rainfall timing, variability, and intensity.

The maps in Figure 7 and Figure 8 suggest that over time, corn may be grown in more northerly areas, and these areas have less sandy soil texture and higher pH soils, which are more suitable for corn growth (Figure 2c,d,g,h). However, the area where Tmax is less suitable expands under future climate change scenarios, and this covers most of southern Georgia for the RCP8.5 scenario, the current area where most corn is grown (compare Figure 3c,g with Figure 7 and Figure 8c,g). Random years with particularly high temperatures and drought could make growing corn in southern Georgia particularly difficult in a warmer future given that high temperatures and drought are currently much more of a problem affecting corn growth in Georgia than cold and wet situations (Figure 6). Southern Georgia is currently where most corn is grown in the state, and in high temperature and drought years crops are particularly susceptible to aflatoxin contamination [46,47]. The FDA legislation surrounding aflatoxin levels can result in whole crop harvests being discarded [34]. This is evidenced by the insurance payments due to aflatoxin contamination of corn shown in Figure 6a. Kerry et al. [46] and Yoo et al. [47] show that June Tmax > 33 °C and June rainfall < 50 mm are associated with a high risk of aflatoxin contamination. Figure 7 and Figure 8c,g and Kerry et al. [36] suggest that June Tmax > 33 °C and June rainfall < 50 mm are more likely to occur in the future. Given the current planting time of corn in Georgia, June corresponds with the fragile mid-silk period of development, and if the crop experiences high temperatures and drought during this period, increased aflatoxin production is more likely.

Given that the 1998–2022 insurance indemnity payments are highest for corn crop loss due to heat and drought (Figure 6), it seems likely that corn crop loss due to heat and drought is likely to increase significantly in a warmer future. This is also suggested by the historical data on % crop loss (Figure 5a). These data show that the 1975–2000 period saw the highest mean and maximum % crop loss. Furthermore, since 2000, the standard deviation of the % crop loss has increased, suggesting greater unpredictability of the weather. Unless the corn growing area shifts further to the north-west, this variability in weather, particularly very hot and droughty conditions and their impact on crops, is likely to continue in the future as climate change shows a warming trend and an increase in extreme weather. Being able to shift the corn growing area in the state of Georgia to the north-west is unlikely to occur, as the Appalachian and piedmont areas of Georgia (Figure 1b) are dominated by the Atlanta urban area and other satellite metro areas which are unlikely to revert back to farmland. Indeed, the original FAO [2] LS classification specifically mentioned the suitability of developing rural rather than urban land. Therefore, given the increasing temperatures in the agricultural south/coastal plain of Georgia, in the future it is likely that rather than shifting production to areas further to the north, the growing season may need to be adjusted, and corn should be planted earlier if the detrimental effects of high summer maximum temperatures are not to be felt. This work also shows the importance of incorporating some aspects of temperature and rainfall timings into LS classifications for the future. Finally, areas that may become more suitable may already be under a different form of land use, which is hard to change. Therefore, strategies other than moving where corn is grown within the state of Geogia need to be sought, such as planting earlier, irrigating to relieve heat stress, using more heat tolerant varieties of corn, or even switching to other crops.

## 4. Conclusions

The LS classification used here, which was adapted from that of Tashayo et al. [8], generally provides a good LS current classification based on significant differences in historic yield, % crop loss, and acres planted between the LS classes. However, these historic data showed less sensitivity to soil factors than temperature factors. This is assumed to be related to the ability to manage soil factors like pH with lime or manage for light soil texture using irrigation. A fully integrated LS classification that includes all layers of information should be developed, but an appropriate method of weighting variables would be needed to account for the ability to manage soil texture and pH limitations but not temperature. The separate analysis of LS classes for different variables was necessary in this work to determine likely future shifts in LS under different climate change scenarios. The work shows that LS classifications for the future may need to be more detailed and that instead of including annual Tmean, Tmin, Tmax, and precipitation, they may need to include growing season values for each of these variables, as the timing of certain temperature and precipitation levels is important. It will also be important to incorporate a notion of degree of variability into future LS classifications, as high standard deviations were observed in the recent historical Tmax, precipitation, and % crop loss data. This work looked at the county level, but future LS classes should be developed for a 1 km grid to account for within-county differences in temperatures, topography, and soils.

This research suggests that the area suitable for corn growing in Georgia could expand under future climate in terms of Tmean and Tmin. However, future Tmax values may pose problems, particularly in southern Georgia, which is currently the main agricultural and corn growing area. This study shows marked shifts in LS class mean centers to the north-west of 100–300 km under climate change scenarios by 2100. As urban areas like the greater Atlanta area dominate in the north of the state, it may not be possible to convert land for agricultural use. This suggests that farmers in southern Georgia may need to plant earlier, irrigate more, or move to other crops relatively soon to avoid more frequent crop failure due to heat and drought or crops being discarded due to high aflatoxin levels. Future LS classifications for the crops that are grown in rotation with corn (soybeans, cotton, and peanuts) [43] should be investigated to see if they show similar patterns and determine if new rotations should be developed. It would also be helpful to develop detailed LS classifications for irrigated corn to determine if the shift in LS would be less if all crops were irrigated and to determine how effective irrigation might be as a mitigation strategy to adjust rainfed corn to the hotter summer conditions that are likely to exist in the future. The findings of this research and future related research could be important to disseminate to local farmers and extension services and to inform agricultural policy going into a warmer future.

## Figures and Tables

**Table 1:** Criteria used to delineate LS zones for rainfed corn (*Zea mays*) in Georgia based on 1991–2020 climate normals (adapted from Tashayo et al. [8]).

Parameters	Highly Suitable	Moderately Suitable	Marginally Suitable	Not Suitable
	(S1)	(S2)	(S3)	(N4)
**Climate factors**				
Annual Tmean (°C)	22–26	18–22 and 26–32	14–18 and 32–35	<14 and >35
Annual Tmin (°C)	16–18	14–16	14–12	<12
Annual Tmax (°C)	24–28	28–32	32–36	>36
**Topog. factors**				
Elevation (m)	<1700	1700–2000	2000–2300	>2300
Slope (%)	0–2	2–6	6–12	>12
**Soil factors**				
pH	6.5–7.5	5.8–6.5 and 7.5–7.8	5.5–5.8 and 7.8–8.2	<5.5 and >8.2
Soil texture	L, CL, SC, C	SL, SCL	LS, ZL, SCL	ZC, S, Z

Tmean, mean temperature; Tmin, minimum temperature; Tmax, maximum temperature. Soil texture classes: C, clay; CL, clay loam; L, loam; LS, loamy sand; S, sand; SCL, sandy clay loam; SL, sandy loam; Z, silt; ZC, silty clay; ZL, silty loam.

## Data Availability

All data used for this research are available from and were generated using freely available data from online repositories and websites, which are given throughout the paper.

## Supplementary Materials

The following supporting information can be downloaded at: https://www.mdpi.com/article/10.3390/plants13172486/s1, Figure S1: (a) Acres of corn planted and (b) corn yield for all counties in the state of Georgia 1954–2020; Figure S2: Maps of historical and future annual mean temperatures (°C) in Georgia; Figure S3: Maps of historical and future annual minimum temperatures (°C) in Georgia; Figure S4: Maps of historical and future annual maximum temperatures (°C) in Georgia; Figure S5: Maps of historical and future annual precipitation (mm) in Georgia.
