# Supplementary material for: Comparing Current and Future Land Suitability for Growing Rainfed Corn (Zea mays) in Georgia, USA
Source: Plants (Basel). 2024 Sep 5;13(17):2486. doi: 10.3390/plants13172486 (PMC11397360; doi:10.3390/plants13172486)
Supplement: Supplementary file 1 [file plants-13-02486-s001.zip › plants-3161481-supplementary.pdf]

plants

Article

Comparing Current and Future Land Suitability for Growing Rainfed Corn (*Zea mays*) in Georgia, USA

Ruth Kerry [1,*], Ben Ingram [2] and Connor S. Golden [1]

[1] Geography Department, Brigham Young University, Provo, UT 84602, USA
[2] Facultad de Ingeniería, Universidad de Talca, Camino a Los Niches Km. 1, Curicó, 3344158, Chile
* Correspondence: ruth_kerry@byu.edu

Supplementary Materials

(a) Acres of Corn Planted 1954-2020 for all Counties in the State of Georgia

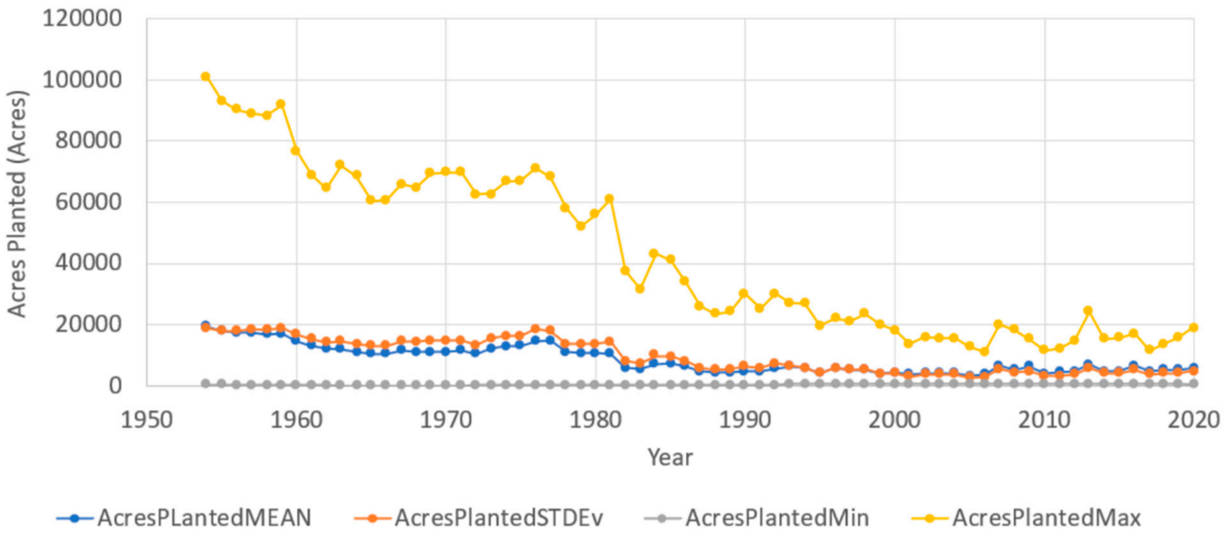

(a) Corn Yield for 1954-2020 for all Counties in the State of Georgia

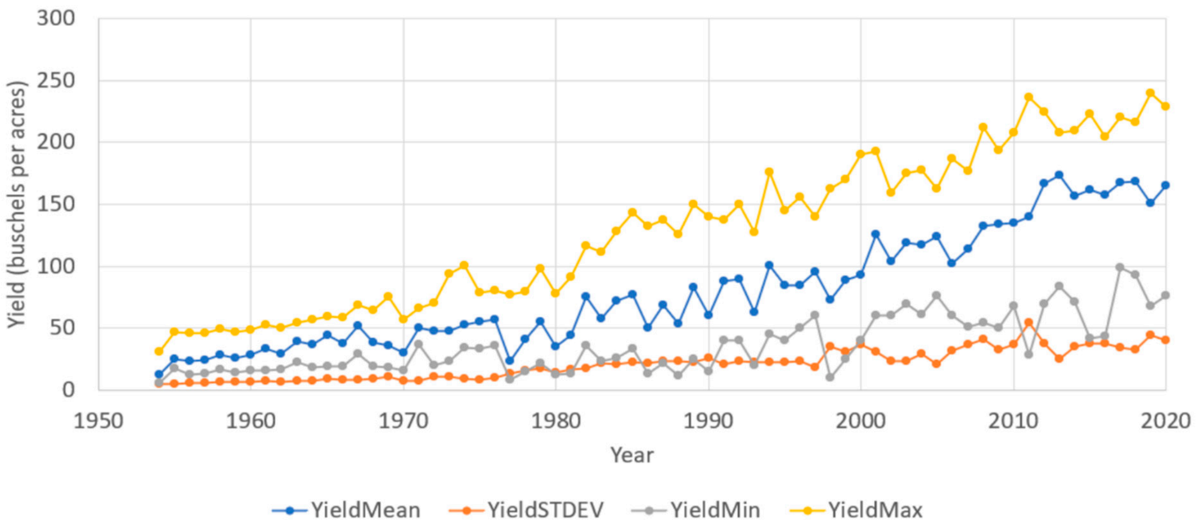

Figure S1. (a) Acres of corn planted and (b) Corn yield for all Counties in the State of Georgia 1954-2020

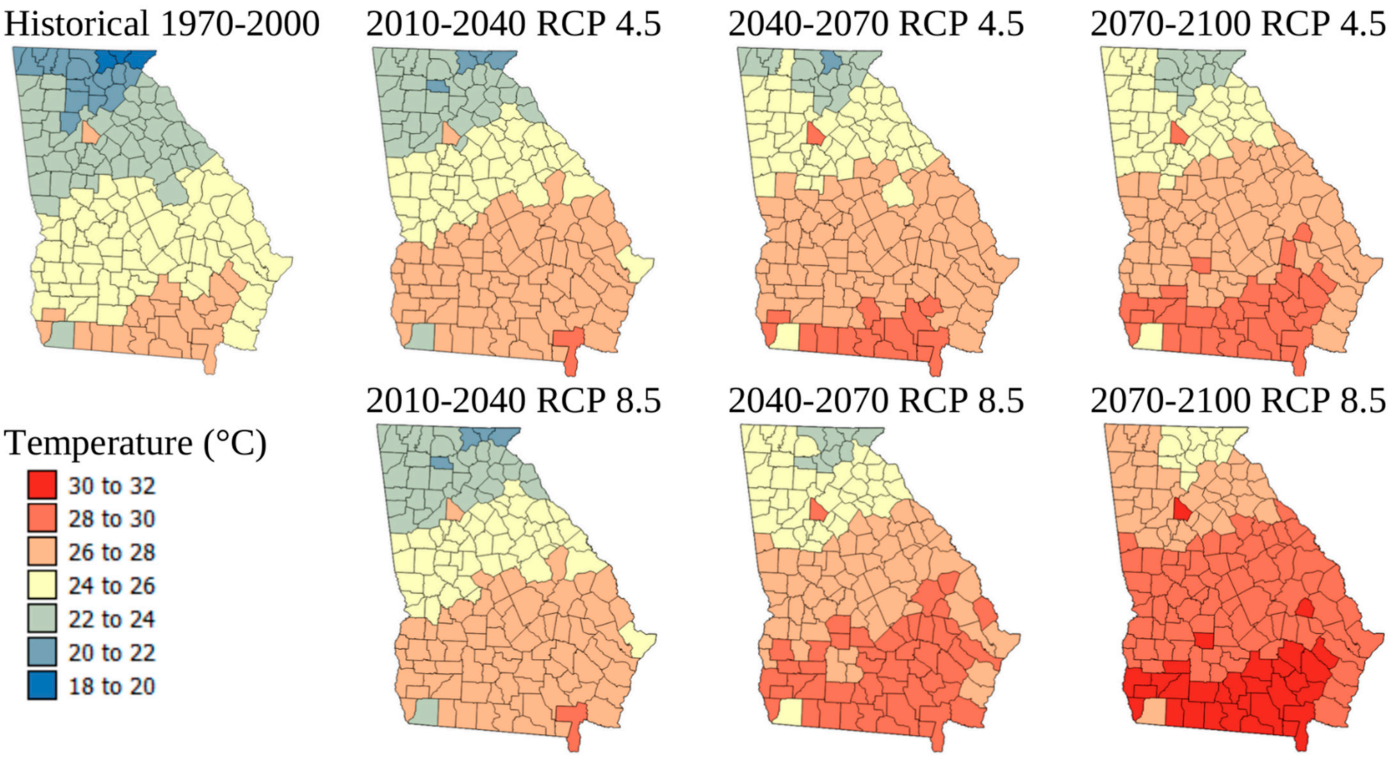

Figure S2. Maps of Historical and Future Annual Mean Temperatures (°C) in Georgia

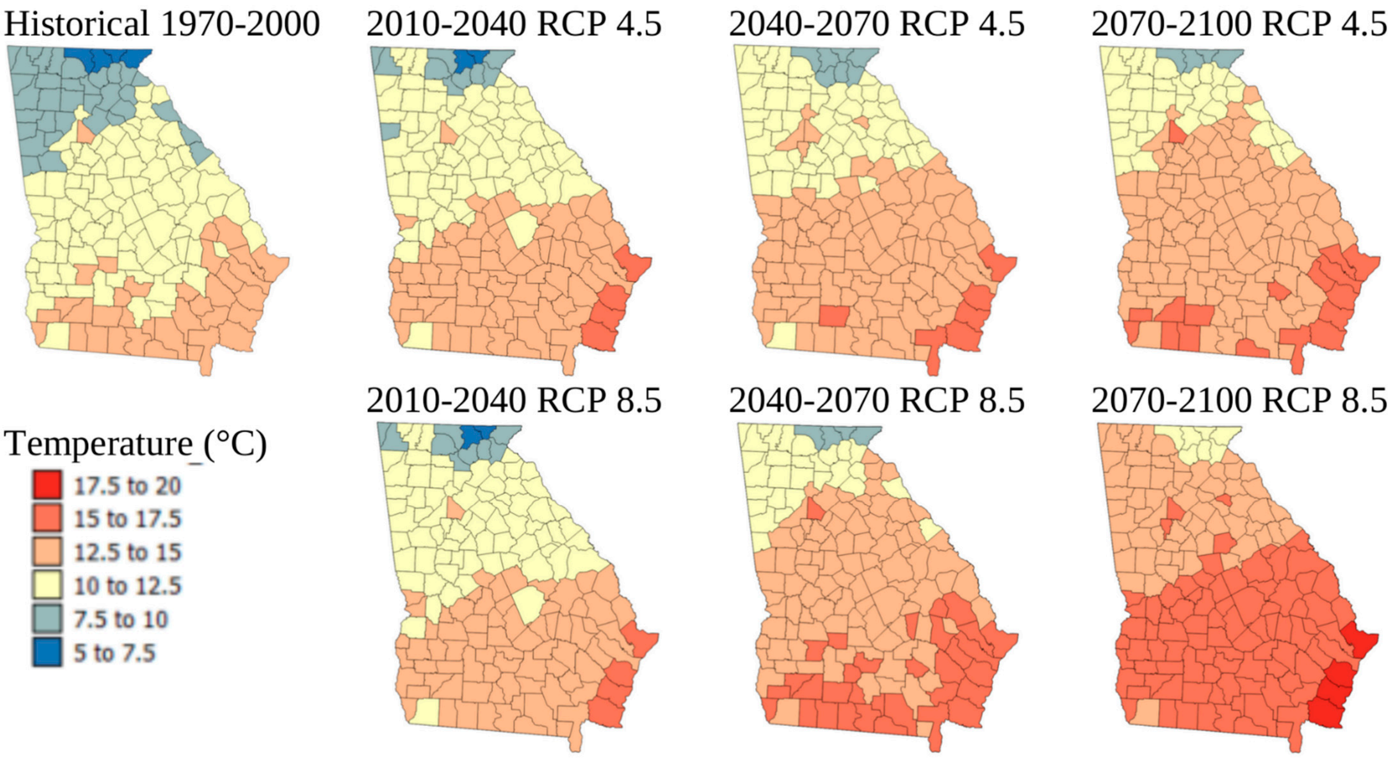

Figure S3. Maps of Historical and Future Annual Minimum Temperatures (°C) in Georgia

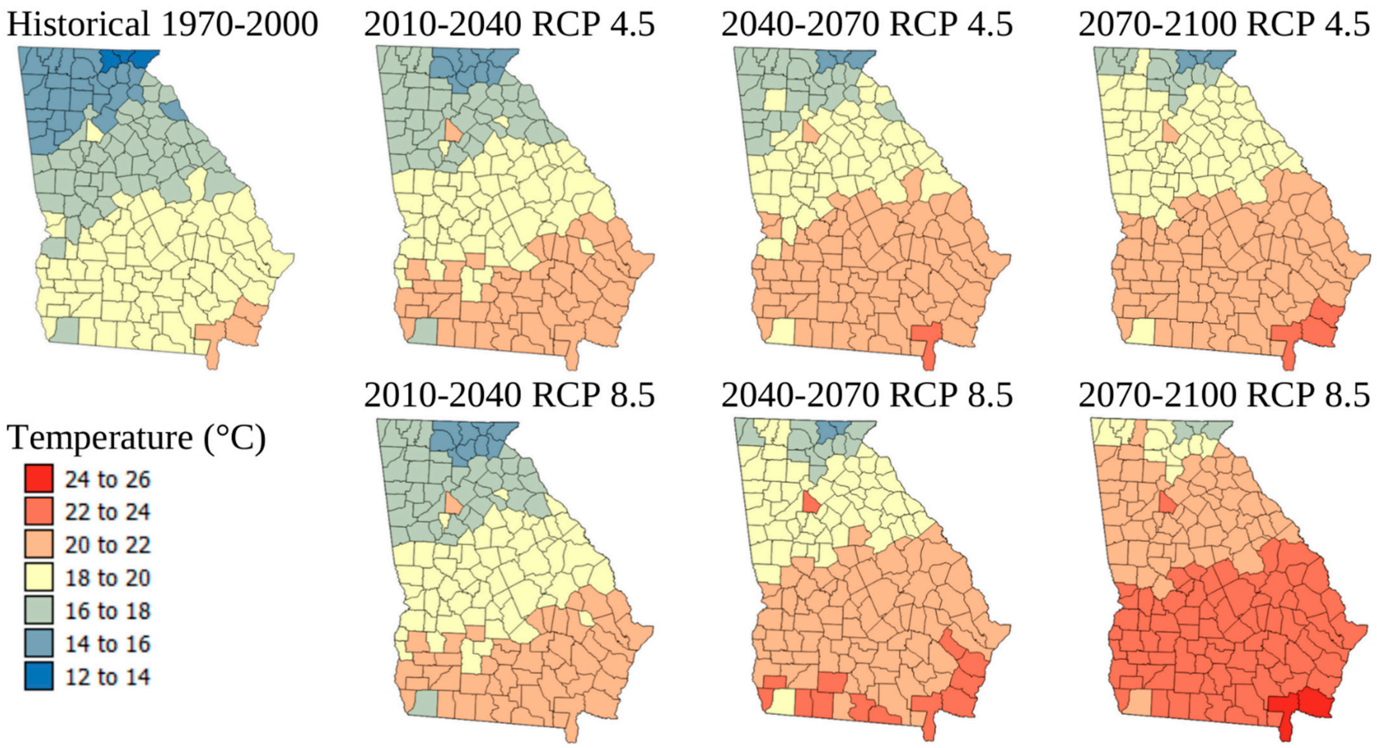

Figure S4. Maps of Historical and Future Annual Maximum Temperatures (°C) in Georgia

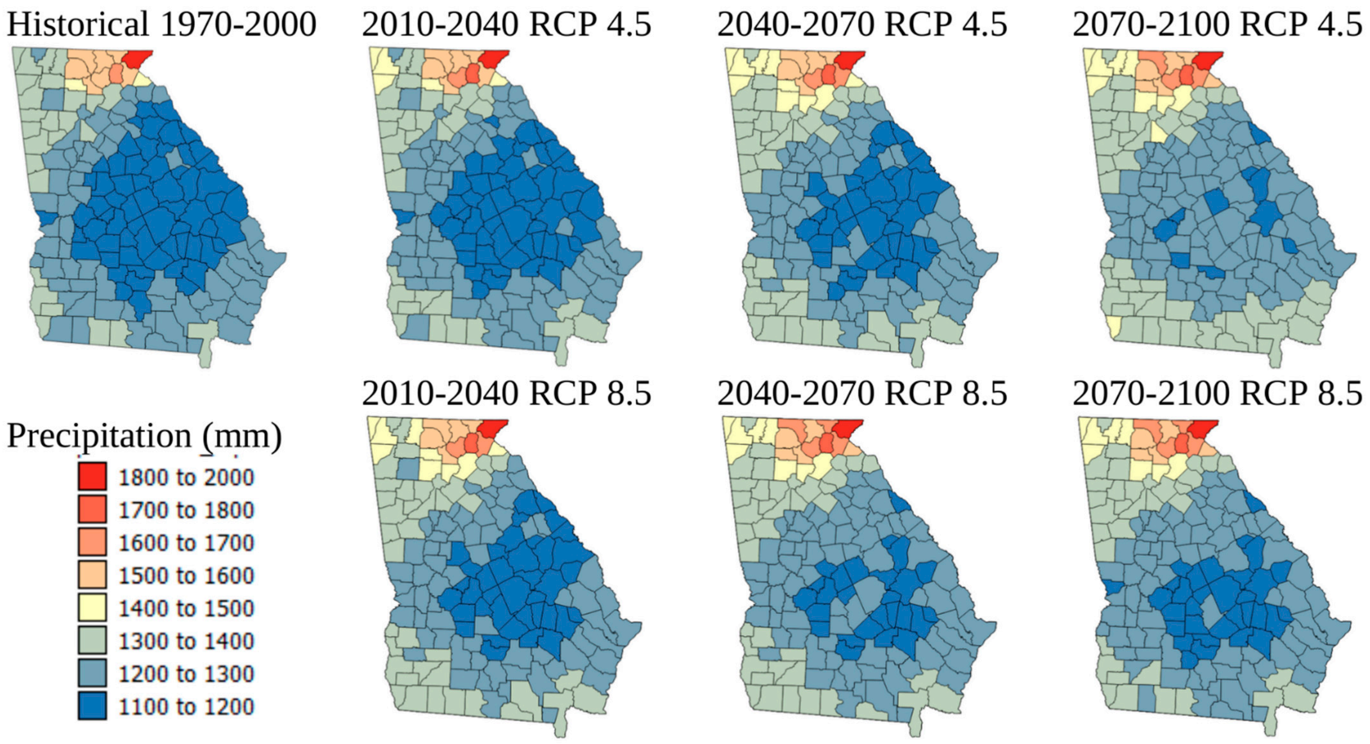

Figure S5. Maps of Historical and Future Annual Precipitation (mm) in Georgia
